# Closely related species differ in their traits, but competition induces high intra‐specific variability

**DOI:** 10.1002/ece3.70254

**Published:** 2024-09-13

**Authors:** Eva Janíková, Marie Konečná, Aleš Lisner, Markéta Applová, Petr Blažek, Anna E‐Vojtkó, Lars Götzenberger, Jan Lepš

**Affiliations:** ^1^ Department of Botany, Faculty of Science University of South Bohemia České Budějovice Czech Republic; ^2^ Department of Functional Ecology Institute of Botany of the Czech Academy of Sciences Třeboň Czech Republic; ^3^ Institute of Entomology, Biology Centre Czech Academy of Sciences České Budějovice Czech Republic

**Keywords:** *Carex* species, closely related species, competition, functional traits, greenhouse experiment, interspecific trait variability, intraspecific trait variability, water availability

## Abstract

Theories explaining community assembly assume that biotic and abiotic filters sort species into communities based on the values of their traits and are thus based on between‐species trait variability (BTV). Nevertheless, these filters act on individuals rather than on species. Consequently, the selection is also influenced by intraspecific trait variability (ITV) and its drivers. These drivers may be abiotic (e.g., water availability) or biotic (e.g., competition). Although closely related species should have similar traits, many of them coexist. We investigated the relative magnitudes of BTV and ITV in coexisting closely related species and how their individual traits differ under different drivers of ITV. We manipulated conditions in a greenhouse pot experiment with four common *Carex* species, where individuals of each species originated from four source localities. Individuals were grown in factorial combinations of two moisture levels, with and without a competitor (grass species *Holcus lanatus,* a frequent competitor). We analyzed the variability of six morphological traits on individuals in the greenhouse and three morphological traits in the source localities. Species identity was the main determinant of differences in most traits. Competition exerted a greater effect than water availability. For leaf dry matter content (LDMC) and vegetative height, competition's effect even exceeded the variability among species. On the contrary, for specific leaf area (SLA) and clonal spread, the interspecific differences exceeded ITV induced by experimental treatments. SLA measured in the greenhouse closely correlated with values measured in field populations, while LDMC did not. The variability caused by source locality of ramets in the greenhouse was small, although sometimes significant. Closely related species differ in their traits, but for some traits, ITV can exceed BTV. We can expect that ITV can modify the processes of community assembly, particularly among coexisting closely related species.

## Graphical abstract

Our study found that closely related species that are similar in their overall appearance and habitus can differ in their functional traits. At the same time, for some traits, phenotypic plasticity (particularly, the part induced by competition) might exceed interspecific differences. Our results point to a potential role of ITV in small‐scale coexistence, in particular for cases where closely related subordinate species respond to competition from dominants.

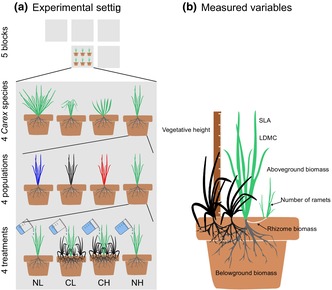

## 1 INTRODUCTION

The trait composition of plant communities is supposed to reflect their functionality, including their response to environmental variation and effect on ecosystem functions (Lavorel & Garnier, 2002). Selection of species from the species pool into a community (Švamberková & Lepš, 2020) is expected to follow assembly rules based on species traits (Götzenberger et al., 2012). Environmental filtering and competitive exclusion of weaker competitors are expected to select for species with similar traits (Carboni et al., 2014; de Bello et al., 2012; Mayfield & Levine, 2010), leading to trait convergence. Contrarily, similar species tend to share niches and thus react to competitors in similar ways, and the concept of limiting similarity (MacArthur & Levins, 1967) suggests that trait divergence is necessary for stable species coexistence. These theories describe the selection of species entering a community and are thus based on interspecific differences. However, selection occurs on the level of individuals, not species (Violle et al., 2012), and thus intraspecific trait differences can considerably modify these processes (He et al., 2021; Lepš et al., 2011; Lisner et al., 2021; Siefert et al., 2015). For example, different responses of individuals within a species to environmental fluctuations can increase a population's persistence (Westerband et al., 2021). Regarding species coexistence, various models predict either positive (by reducing interspecific relative to intraspecific competition) or negative effects of intraspecific trait variability (i.e., differences within species; ITV) on population persistence. But it is likely that both mechanisms act simultaneously (Westerband et al., 2021). Many studies suggest that interspecific trait variability (i.e., between‐species trait variability; BTV) is generally much higher than ITV (Garnier et al., 2001; McGill et al., 2006). Nevertheless, ITV can be as important as BTV (Albert et al., 2010; Messier et al., 2010) or even more important than BTV (Clark et al., 2004). This is especially the case for habitats with high micro‐environmental heterogeneity at the local or community scale (Westerband et al., 2021). In addition to the magnitude of ITV, we should also consider whether the environment affects trait values of different species similarly (technically, no interaction of species with environment). In this case, species rankings according to their trait values will be constant, suggesting constant functional differences among species in different environments (Shipley et al., 2016). Garnier et al. (2001) and Mudrák et al. (2019) indeed found no, or only a few, significant interactions indicating a change in species ranking for a wide range of species. Nevertheless, also in the case of constant functional differences, the consideration of ITV can significantly improve conclusions about community assembly processes (He et al., 2021; Jung et al., 2014; Shipley et al., 2016).

Intraspecific trait variability (ITV) can result from genetic differences, epigenetic variation (Latzel et al., 2012; Puy et al., 2021) and phenotypic plasticity (Albert et al., 2011; Jung et al., 2014). Phenotypic plasticity is the result of changes in environmental conditions, both abiotic and biotic (March‐Salas et al., 2020), which influence species survival (Švamberková & Lepš, 2020) and their functional traits (Jessen et al., 2020; Valladares et al., 2007; Violle et al., 2012). Water regime (Li et al., 2020) and competitive pressure (Hao et al., 2023) are two important factors potentially affecting plasticity of different traits with varying intensities. For example, physiological or chemical traits are considered to be more plastic in different environments than morphological traits (Dostál et al., 2020; Ji et al., 2020; Westerband et al., 2021). Vegetative height, specific leaf area (SLA), and leaf dry matter content (LDMC) are among both the most reported and most frequently used traits for understanding functional variation across species. At the same time, these traits are supposed to be rather constant with low ITV, although in general ITV tends to be greater for plant vegetative height relative to leaf traits (Siefert et al., 2015). Maximum vegetative height represents the competitive ability of species (Pérez‐Harguindeguy et al., 2016). SLA and LDMC are associated with the leaf economic spectrum (Wright et al., 2004) reflecting the trade‐off between acquisitive and conservative strategies. Clonal traits and Root/Shoot ratio tend to be rather plastic traits exhibiting considerable ITV (Siefert et al., 2015; Zhang et al., 2023). Clonal traits, such as lateral spread (horizontal distance to which a clonal growth organ can spread per year), multiplication rate (number of clonal offspring produced by a mother ramet per year), and persistence of rhizomes (number of years of rhizome survival), reflect the capacity for vegetative reproduction and horizontal growth (Klimešová et al., 2021). Water stress generally decreases species aboveground biomass, vegetative height, and SLA, and increases root biomass and LDMC (Li et al., 2020; Wilson et al., 1999). It is, however, unclear how clonal traits would respond. High resource supply should support higher branching intensity (de Kroon & Hutchings, 1995), while negative biotic interactions (e.g., competition, herbivory) can moderate horizontal growth (Cornelissen et al., 2014). Overall, environmental variability (e.g., water availability gradient) can play a more important role in the presence of competition than in areas with no or low competitive pressure. For example, in Janíková and Lepš (2023), the survival of species was not affected by water gradient in competition free gaps but was affected in plots with competition. Likewise, trait differences can be more affected by abiotic factors if species are exposed to competition (Hao et al., 2023). It is, however, not sufficiently understood to what extent the total ITV is caused by genetic and epigenetic factors rather than by abiotic stress and biotic interactions. In the field, we are not able to distinguish individual factors influencing ITV. Differences among individuals of the same locality are generally considered “random variability” (i.e., the cause of differences remains unclear). Differences among local populations (localities) might be determined both genetically (incl. epigenetic factors) and by any of the (measured or unmeasured) environmental characteristics of the locality. Common garden experiments (e.g., pot experiments using individuals from various source localities and with experimentally varying conditions) can be used to disentangle the effects of individual possible sources of ITV (de Bello et al., 2021; Gorné et al., 2021).

*Carex* is one of the largest genera among Angiosperms (Reznicek, 2001) with a nearly cosmopolitan distribution and the highest diversity in the boreo‐temperate zone (Martín‐Bravo et al., 2013). These species can be morphologically variable and differ considerably in some of their functional traits, despite their relatedness (Jiménez‐Mejías et al., 2016). The genus is also very diverse in Central Europe, with more than 90 species in the Czech Republic (Kaplan et al., 2019). In Central Europe, many *Carex* species prefer wet habitats, particularly meadows, and often co‐occur (Lepš et al., 2018). However, although the coexisting species undoubtedly compete with each other, the meadow *Carex* species seldom achieve high cover and dominance, but rather coexist in a matrix dominated by grasses. The response of *Carex* species (typically subordinate species in meadows) to competition with dominant grass species can therefore be crucial for their coexistence (Grime, 1998). Closely related species (e.g., *Carex* spp.) are expected to have similar trait values, if these traits are phylogenetically conserved (Davies et al., 2013; Schmidt et al., 2018). The question then is whether BTV of closely related species exceeds ITV, and whether these species' traits react to abiotic (e.g., water availability) and biotic (e.g., competition) factors in the same manner (resulting in no interaction of species identity with the treatment of interest). It is necessary to point out that there are differences among individual traits—some of them are rather species‐specific (e.g., plant vegetative height, SLA, and LDMC), whereas in others the response to environmental variation is more pronounced (e.g., leaf nutrients) (Mudrák et al., 2019).

We conducted a greenhouse experiment with four closely related *Carex* species, from four different populations each, and measured their functional traits under water availability and competition treatments. We were interested in the size of intraspecific differences caused both by experimental treatments (competition, water availability) and by the origin of individual transplants (source locality), as well as how ITV compares to BTV of these species. We also compared traits measured in a greenhouse with traits measured in the source localities to disentangle the effect of individual factors (genetic versus environmental) influencing ITV. We aimed to answer the following questions to guide our experiment: (1) How are the traits of individual species affected by competition and water availability, and how does this induced ITV compare with their interspecific differences? (2) Do closely related species respond similarly to competition and water availability? (3) How well are the functional trait differences in nature (among and within species) reflected in the trait values of the greenhouse experiment? We expected significant interspecific differences for individual functional traits, even for these closely related species (all species belonging to the subgenus *Carex* within the *Carex* genus) because, although these species can coexist, they also occur separately (they have different optima for soil moisture). We expected high effects of both competition and water availability on traits for different *Carex* species and possible, although weak, interactions between species and treatment and also some possible effect of the species' source locality. All the above effects were expected to be rather trait specific, and we aimed to see which of them had the most stable BTV. However, we did not expect that differences in traits among populations of the same species from nature would be closely reflected by differences measured in the greenhouse experiment because genetic factors are the main drivers of ITV in standard greenhouse conditions, while ITV is mainly driven by environment in the field.

## 2 MATERIALS AND METHODS

### 2.1 Species and localities

For our greenhouse experiment, we chose four common *Carex* species (Table S1 in Appendix S1) able to co‐occur, although they differ especially in preference of soil moisture (Ellenberg‐type indicator value for moisture is 4 for *Carex caryophyllea*, 5 for *Carex pilulifera*, 6 for *Carex pallescens* and 8 for *Carex panicea*; Chytrý et al., 2018). These species are all perennial herbs, hemicryptophytes, commonly distributed in the South Bohemia region, where they were recorded in all 12 × 11 km quadrats of grid mapping and in the majority of their 6 × 5.5 km subquadrats (Pladias—database of Czech flora and vegetation, www.pladias.cz, access June 13, 2024; Wild et al., 2019). These species are able to co‐occur, although they also often occur separately, especially in the case of *C. caryophyllea* which prefers drier habitats. Although these *Carex* species frequently co‐occur in relatively small plots (2 × 2 m, personal observation), they seldom achieve high cover, and the main competitor is commonly some dominant grass. The average cover of our *Carex* species in phytocenological relevés from Pladias—the database of Czech flora and vegetation (only relevés where the species is present considered) is relatively low (3.6% *C. caryophyllea*, 7.9% *C. panicea*, 2.4% *C. pallescens*, 2.5% *C. pilulifera*) with a maximum recorded cover of 38% (i.e., degree 3 of the Braun‐Blanquet scale) for all species but *C. panicea*, which can become dominant (but only rarely and in special types of peatland meadows) (www.pladias.cz, access June 13, 2024; Chytrý et al., 2021). We used populations from four source localities (size 1–3 ha) of the South Bohemia region for each species. One locality contained all four species in mixtures, demonstrating they can coexist; together we used individuals from seven localities (Table S1 in Appendix S1). All localities were semi‐natural meadows with regular mowing management, mostly once per year with the exception of one locality, which was not managed. From each population, we took 20 individuals in the form of small ramets (young vegetative rosettes, average fresh weight = 0.58 ± 0.43 g (mean ± SD), average vegetative height = 5.05 ± 2.82 cm) at the beginning of April 2020 for the greenhouse experiment.

### 2.2 Design of greenhouse experiment

**FIGURE 1:**
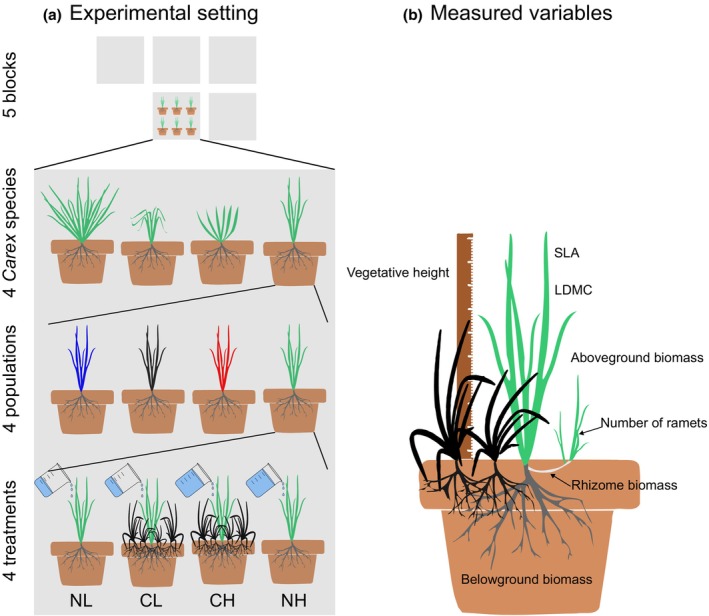
Experimental setting (a) and measured variables (b) of greenhouse experiment. CL, competition and low water treatment; CH, competition and high water treatment; NH, no‐competition and high water treatment; NL, no‐competition and low water treatment.

Each individual was taken from the field to the greenhouse and left in water to develop roots for 1 week. After that, we planted each individual in its own pot (upper diameter 16 cm, lower diameter 10 cm, height 15 cm, volume 2 L) with peat substrate and sand mixture in the ratio of 1:2. From each population, 20 ramets were grown placing them randomly in five blocks in factorial combinations of two moisture levels (water treatment), and with and without competition of *Holcus lanatus* (competition treatment) (Figure 1a). Thus, we had four experimental treatment type combinations: CH—competition and high water treatment, CL—competition and low water treatment, NH—no‐competition and high water treatment, and NL—no‐competition and low water treatment. In all our source localities (and similar localities in the area), sedges grew in the matrix of grasses, which dominate the meadows and thus are their main competitors. *Holcus lanatus* (Ellenberg‐type indicator value for moisture is 6; Chytrý et al., 2018) is their typical representative. It is a clonal hemicryptophyte, typically with a height similar to *Carex* species from our experiment. It is one of the most frequently co‐occurring species with *Carex* in our source localities; it is also suitable for experiments, as its germination rate is quite high and it establishes quickly (Tammaru et al., 2021). We used seeds from a commercial supplier (Planta Naturalis). For the competition treatment, ca. 45 seeds of *Holcus lanatus* were sown in each pot together with the *Carex* ramet at the time of ramet planting. After 2 weeks, *Holcus lanatus* was thinned to 15 individuals per pot. One week after thinning, when *Holcus lanatus* was established, the water treatment was started. While the high water treatment consisted of keeping the saucer below the pot (volume of 380 mL) permanently filled with water, in the low water treatment meant saucers were only filled with water once the substrate was dried out. Because we could not measure the soil water content directly in every pot, we checked the effect of our water availability treatment on *Carex* biomass aboveground (18% decrease in low water treatment, *F*_1,312_ = 7.43, *p* = .007), belowground (24% decrease in low water treatment, *F*_1,309_ = 19.76, *p* < .001) and in total (22% decrease in low water treatment, *F*_1,307_ = 14.95, *p* < .001) to prove that the low water treatment was sufficient to impose water stress. Because we used nutrient‐poor substrate (peat with sand), with fertility insufficient for *H. lanatus*, we used 100 mL of fertilizer (15 mL of Vitality Komplex Agro on 1 L of water) on each pot two times. All plants were harvested from July 29 to August 1, 2020. They were washed free of soil, separated into aboveground (leaves, stems, and flowers) and belowground (roots and rhizomes) parts, and processed for measuring functional traits as described below.

### 2.3 Measured functional traits and other variables

In the greenhouse experiment, we measured six functional traits characterizing different functions of *Carex* plants (Figure 1b). Vegetative height was measured as the highest leaf apparently forming a rosette without stretching. To calculate SLA and LDMC, we took the fresh and dry weights of two leaves per individual and measured their area in ImageJ 1.× (Schneider et al., 2012). The number of ramets created by the mother plant in each pot (Clonal spread further on) determined the ability of clonal multiplication. Belowground and aboveground biomass (with total biomass the sum of above‐ and belowground biomass) from each pot was oven‐dried at 60°C for 48 h and subsequently weighed. From these values, we calculated the Root/Shoot ratio (i.e., dry biomass of only roots divided by dry aboveground biomass) and Aboveground/Belowground biomass (i.e., dry aboveground biomass divided by dry belowground biomass of both roots and rhizomes) to differentiate belowground biomass as only roots, or including rhizomes, and to compare them with the aboveground biomass.

To compare functional traits measured on plants grown under specific greenhouse conditions and experimentally influenced by water and competition treatments with traits measured in the source localities, we also measured three functional traits (Vegetative height, SLA and LDMC) of 10 individuals per population sampled from the field (i.e., field study) at the same time the greenhouse experiment was harvested, selected in the field according to Pérez‐Harguindeguy et al. (2016).

### 2.4 Data analysis

#### 2.4.1 Inter‐ and intraspecific trait variability

**FIGURE 2:**
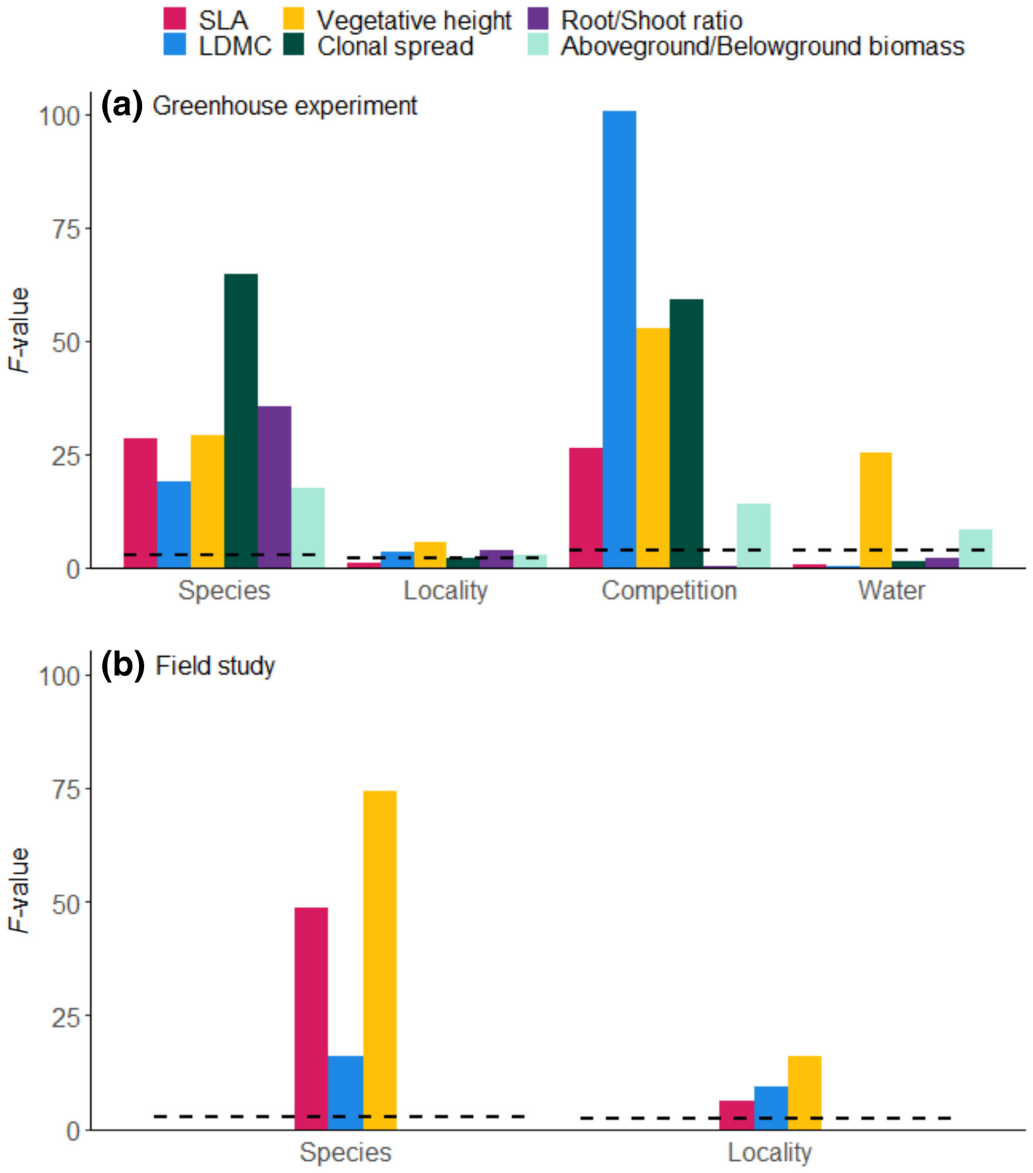
Effect size (value of *F* test from ANOVA for LMM) of model terms for individual traits measured in greenhouse experiment (a) and in field study (b). Dashed black line represents the critical *F*‐values for appropriate degrees of freedom (see Table 1 in the main text) and *α* = 0.05.

We used Linear Mixed‐Effects Models (LMM) with *F* tests of type III analysis of variance (ANOVA) using R version 4.4.0 (R Core Team, 2021), package “lmerTest” (Kuznetsova et al., 2017) to explain measured values of individual traits in the greenhouse experiment based on species identity (Species), source locality (Locality), experimental treatment (Competition and Water) and all their interactions. These factors were taken as fixed with experimental block (Block) as random effect because Block had significant effect on three out of six tested traits (Table S2 in Appendix S1). For this analysis, after a check of the data normality, we used Log‐transformed data for SLA, Vegetative height, Root/Shoot ratio and Aboveground/Belowground biomass and Square Root‐transformed data for Clonal spread. The same test was used for aboveground, belowground and total biomass of *Carex* (all values Log‐transformed) to check the effectivity of the water treatment. To examine BTV and ITV for plants from the field study, we used values measured in the field of Vegetative height (Log‐transformed), SLA, and LDMC in linear models (LM) with an *F* test of ANOVA, where we tested the effect of Species, Locality, and their interaction. For graphical representation (Figure 2), we calculated critical values for *F* (for individual factors both for greenhouse experiment and field study) reflecting appropriate degrees of freedom (df) of each factor and *α* = .05.

Because we wanted to compare the explained variability of the greenhouse experiment with that of the field study represented by Sum of Squares, we reanalyzed the LMM for the greenhouse experiment without the random factor Block and using a LM with *F* test of ANOVA, as in the case of the field study. The effect of Block, which was not included in this new analysis, was thus included in residuals of the model. Explained variability was composed by the Sum of Squares of Species, Locality, Experiment (i.e., Competition, Water and their interaction; only for greenhouse experiment), Interactions (i.e., all interactions with Species and Locality; because the number of tested interactions was high, we summed up the Sum of Squares of all interactions with Species and Locality together for clarity) and Residuals (i.e., variability explained by other factors that were tested in the model, including the effect of Block for the greenhouse experiment). While the variability among Species expressed BTV, ITV included variability of Locality, Experiment and Interactions. The variability of individuals from the same population (the same species from the same locality) under the same treatment combination is reflected by the Residuals (including the unexplained ITV, as well as sampling and measurement errors).

Although one of our aims was to compare BTW and ITV, we should be aware that this comparison is context dependent and also depends on the design of the experimental setup. In particular, the explained variability depends on the degrees of freedom (generally, the explained variability increases with the degrees of freedom used). Nevertheless, the explained variability (expressed using Sum of Squares) is additive, which is a great advantage. Also, because the same design and data analysis model was used for all the traits, it is ideal for comparison of variability explained by individual factors in various traits. For direct comparison of effects of individual factors (i.e., species identity, individual experimental factors, source locality), the simple *F*‐value might be more useful as a measure of the effect size. It is the ratio of variability explained in given experimental settings by an individual factor to its expected value under the null hypothesis that it does not have any effect. The disadvantage of *F*‐value is that it is not additive.

#### 2.4.2 Correlations of traits between greenhouse experiment and field study

To assess how well the functional trait differences among and within species in nature were reflected in the values of their traits in the greenhouse experiment, we calculated the average values of functional traits from the field and greenhouse for each population and four experimental treatment type combinations (CH, CL, NH, NL). Subsequently, we compared the values from the field with values from the greenhouse using Major axis regression (MAR) in R‐package “lmodel2” (Legendre, 2018). We also calculated between group (i.e., between‐species) and within group (i.e., within‐species between localities) Pearson's correlation coefficients between trait values from the field and greenhouse using function “statsBy” from the R‐package “psych” (Revelle, 2024), where the grouping variable was *Carex* species. Nevertheless, especially in the case of between group correlations, the statistical test was not very relevant because it was calculated only from four values (i.e., four *Carex* species), which cannot give significant values for such a dataset. Thus, it was rather used to identify trends without testing for statistical significance.

For all figures, we used R‐package “ggplot2” (Wickham, 2016) and represented all data as non‐transformed for easier visual interpretation.

## 3 RESULTS

### 3.1 Inter‐ and intraspecific trait variability

**FIGURE 3:**
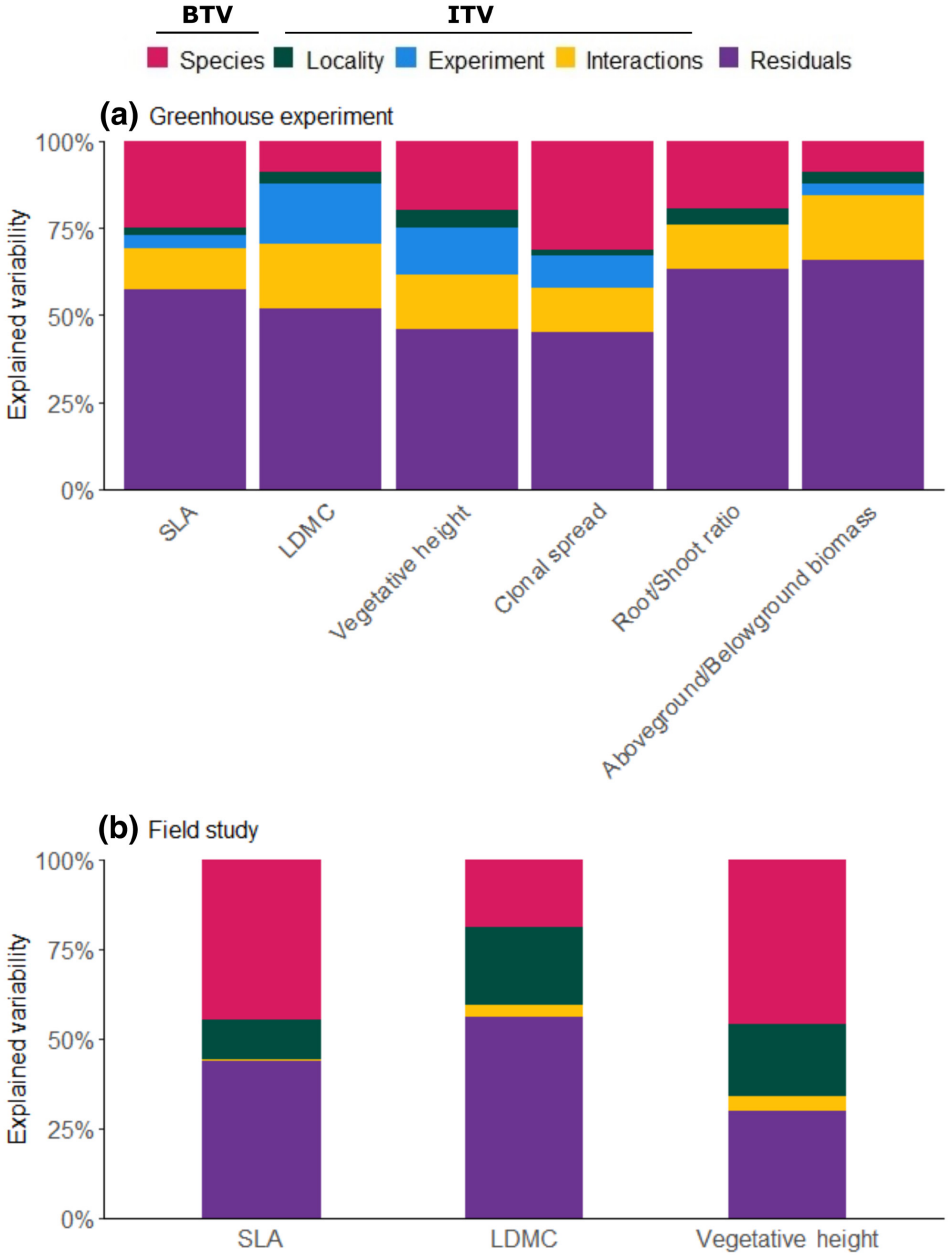
Explained variability by Species (species identity), Locality (source locality), Experiment (i.e., the effect of competition, water availability, and their interaction), Interactions (i.e., all interactions with Species and Locality), and Residuals (i.e., variability explained by other factors than those that are tested in the model) for individual traits in greenhouse experiment (a) and in field study (b). BTV, between‐species trait variability; ITV, intraspecific trait variability.

All functional traits measured both in the greenhouse and in the field differed significantly among the studied species (Table 1, Figure 2, Table S3 in Appendix S1). The traits were influenced not only by species identity, but also by some experimental treatments (competition and water availability) and sometimes by the effect of source locality (Figure 2). When measured by the explained variability (i.e., Sum of Squares), the model explained more ITV than BTV for LDMC, Above/Belowground biomass and Vegetative height in the greenhouse experiment (Figure 3a) and for LDMC in the field study (Figure 3b). For Root/Shoot ratio, BTV and ITV explained by the model were fairly equal (Figure 3a). The model explained less ITV than BTV for SLA (in both greenhouse experiment and field study), Clonal spread in the greenhouse experiment, and Vegetative height in the field (Figure 3).

**TABLE 1:** Results of ANOVA for LMM of individual functional traits measured in greenhouse experiment.

	SLA			LDMC			Vegetative height			Clonal spread			Root/Shoot			Aboveground/Below‐ground biomass		
	df	*F*	*p*	df	*F*	*p*	df	*F*	*p*	df	*F*	*p*	df	*F*	*p*	df	*F*	*p*
Species	**3, 305**	**28.40**	**<.001**	**3, 306**	**18.71**	**<.001**	**3, 316**	**29.23**	**<.001**	**3, 312**	**64.78**	**<.001**	**3, 308**	**35.43**	**<.001**	**3, 312**	**17.57**	**<.001**
Locality	6, 305	1.01	.418	**6, 306**	**3.26**	**.004**	**6, 316**	**5.42**	**<.001**	6, 312	2.12	.050	**6, 308**	**3.72**	**.001**	**6, 312**	**2.53**	**.021**
Competition	**1, 305**	**26.19**	**<.001**	**1, 306**	**100.46**	**<.001**	**1, 316**	**52.59**	**<.001**	**1, 312**	**59.11**	**<.001**	1, 308	0.20	.655	**1, 312**	**13.85**	**<.001**
Water	1, 305	0.52	.471	1, 306	0.06	.810	**1, 316**	**25.31**	**<.001**	1, 312	1.11	.294	1, 308	1.98	.160	**1, 312**	**8.15**	**.005**
Species × Locality	6, 305	0.37	.898	6, 306	1.72	.116	**6, 316**	**2.75**	**.013**	**6, 312**	**4.35**	**<.001**	6, 308	0.38	.893	6, 312	1.38	.223
Species × Competition	3, 305	1.59	.192	**3, 306**	**3.59**	**.014**	**3, 316**	**3.98**	**.008**	3, 312	0.86	.464	3, 308	2.09	.102	**3, 312**	**3.57**	**.014**
Locality × Competition	6, 305	1.81	.097	6, 306	1.59	.150	6, 316	0.58	.746	6, 312	1.67	.127	6, 308	1.11	.354	6, 312	1.30	.258
Species × Water	3, 305	2.59	.053	**3, 306**	**5.01**	**.002**	3, 316	1.51	.211	3, 312	0.10	.966	**3, 308**	**3.17**	**.024**	**3, 312**	**3.21**	**.023**
Locality × Water	6, 305	1.52	.171	**6, 306**	**3.41**	**.003**	**6, 316**	**4.17**	**<.001**	6, 312	0.92	.479	**6, 308**	**3.64**	**.002**	**6, 312**	**3.49**	**.002**
Competition × Water	1, 305.	3.75	.054	**1, 306**	**6.92**	**.009**	1, 316	3.45	.064	1, 312	0.53	.468	1, 308	0.70	.403	1, 312	0.39	.530
Species × Locality × Competition	6, 305	1.18	.318	6, 306	1.22	.298	6, 316	1.28	.267	6, 312	0.46	.837	6, 308	0.4	.877	6, 312	1.03	.408
Species × Locality × Water	6, 305	1.23	.289	6, 306	1.63	.138	**6, 316**	**2.98**	**.008**	**6, 312**	**2.60**	**.018**	6, 308	1.06	.387	6, 312	1.67	.129
Species × Competition × Water	3, 305	1.77	.153	3, 306	1.55	.202	3, 316	1.66	.175	3, 312	0.71	.544	3, 308	2.06	.106	3, 312	2.18	.091
Locality × Competition × Water	**6, 305**	**2.81**	**.011**	6, 306	1.33	.244	6, 316	0.51	.804	6, 312	0.81	.567	6, 308	1.01	.418	6, 312	0.74	.611
Species × Locality × Competition × Water	6, 305	0.52	.800	6, 306	2.05	.059	6, 316	1.00	.428	**6, 312**	**3.49**	**.002**	6, 308	0.50	.807	6, 312	0.84	.541

*Note*: Statistically significant results (*p* < .05) are given in bold.

**FIGURE 4:**
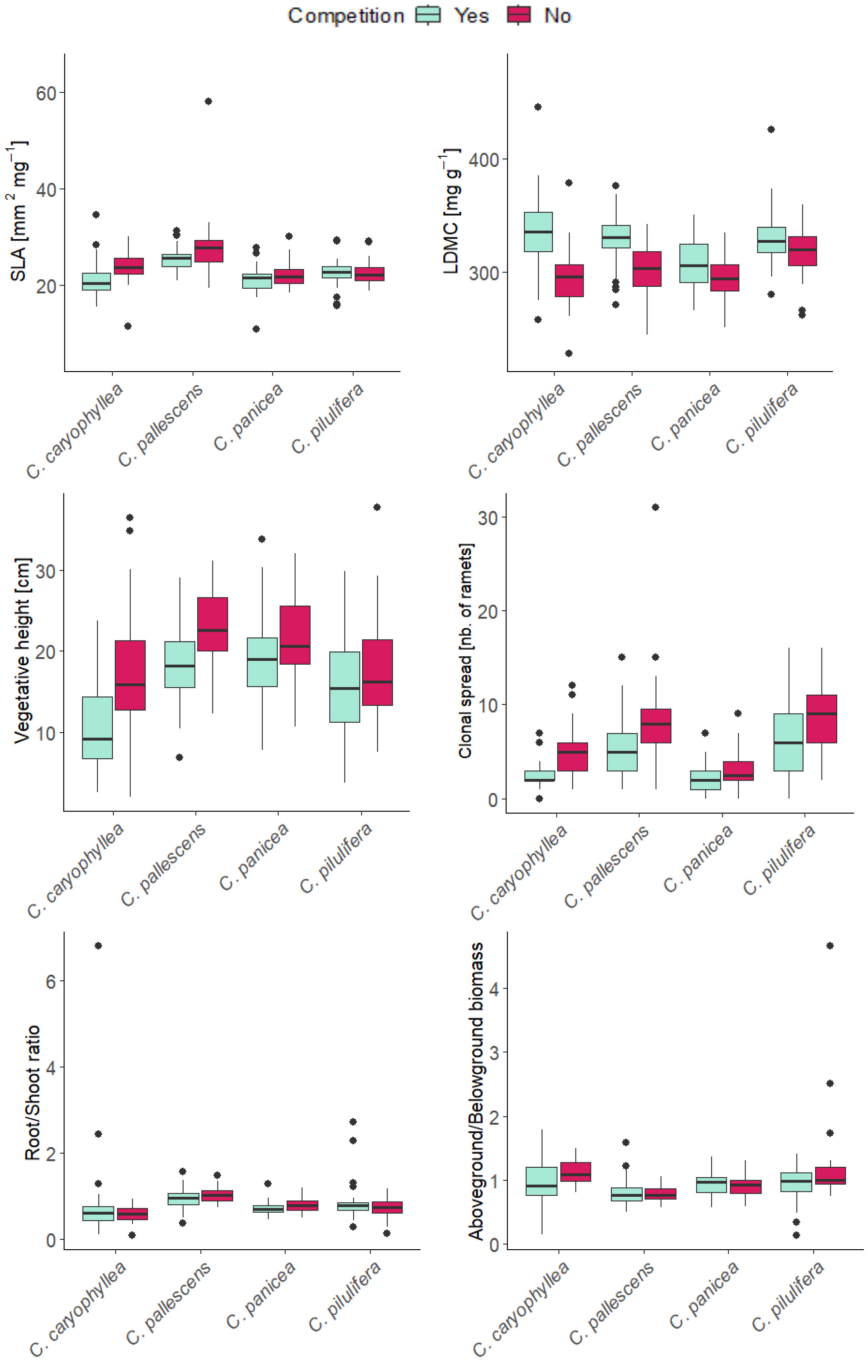
Species × competition interaction of functional traits measured in greenhouse experiment. Results of statistical test are in Table 1.

Of the two experimental treatments, competition was more important than water availability and significantly influenced all traits, with the exception of Root/Shoot ratio (*F*‐value used for direct comparison of effects of experimental treatments; Table 1, Figure 2a). In the case of LDMC and Vegetative height, the effect of competition was even higher than the effect of species (Table 1, Figure 2a), contributing greatly to the overall high ITV component for those two traits. Competition decreased SLA, Vegetative height, and Clonal spread, while it increased LDMC (Figure 4). For LDMC, Vegetative height and Aboveground/Belowground Biomass measured in the greenhouse, the effect of competition differed among species (significant interaction, Table 1, Figure 4). Water availability significantly positively affected only Vegetative height and negatively affected the ratio of Aboveground/Belowground biomass (Table 1, Figure 2a). We recorded a significant interaction between species and water availability for LDMC, Root/Shoot ratio, and Aboveground/Belowground biomass (Table 1, Figure S1 in Appendix S1). The only significant interaction between competition and water availability was for LDMC (Table 1). Without competition, high water treatment increased the LDMC in comparison with low water treatment. With competition, surprisingly, there were no differences in LDMC caused by water availability (Figure S2 in Appendix S1).

The effect of the source locality on functional traits measured in the greenhouse was significant for LDMC, Vegetative height, Root/Shoot ratio, and the ratio of Aboveground/Belowground biomass (*F*‐value used for direct comparison of effects of source locality; Table 1, Figure 2a), although for all these traits the effect of source locality was much smaller than that of species (Table 1, Figure 2a). For traits measured in the field, the effect of source locality was significant for all three traits (Table S3 in Appendix S1) and higher than in the case of greenhouse measurements (Figure 2).

### 3.2 Correlations of traits between greenhouse experiment and field study

**FIGURE 5:**
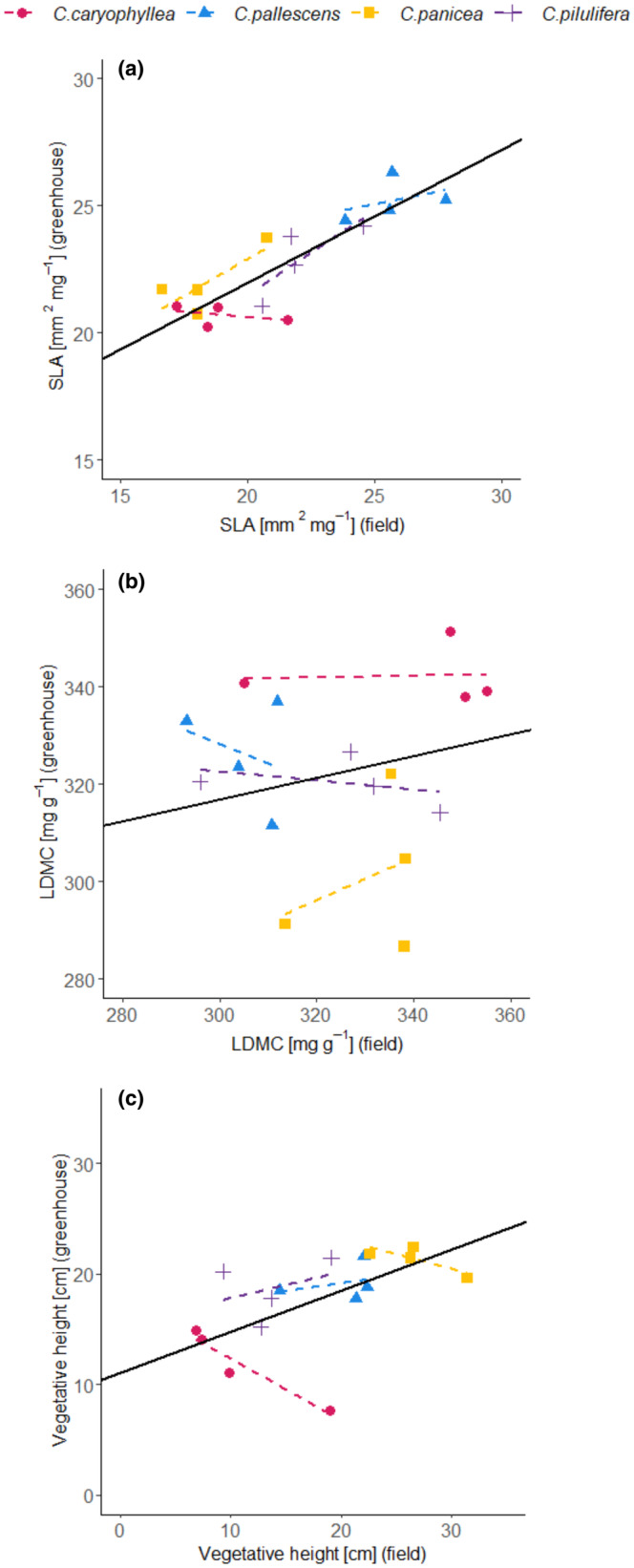
Major axis regression of functional traits measured in the field and greenhouse (competition and high water treatment). Solid line: Overall trend from Major axis regression. Dashed lines: Trend within‐species between localities. Results of statistical test are given in Table 2.

Values of SLA measured in greenhouse populations correlated quite well with its values measured in field populations, while values of LDMC did not correlate at all (Major axis regression in Table 2, Figure 5a,b) in the case of all treatment combinations. Values of Vegetative height from greenhouse populations marginally correlated with values from field populations in most of treatment combinations (Table 2, Figure 5c). Between‐species correlations were always higher than within‐species between locality correlations for SLA and Vegetative height, while for LDMC the differences were not so evident (Table 2). For SLA, the between‐species correlations were the highest, especially in the case of the treatments with competition (Table 2). The trends within species only sometimes reflected the overall trend (Figure 5). However, the more consistent the trait (i.e., high correlation coefficient between greenhouse and field populations), the more similar within‐species reflection of the overall trend, although this was quite dependent on species (Figure 5) and treatment combination (Table 2).

**TABLE 2:** Pearson's correlation coefficients (*r*) and probabilities (*p*) for Major axis regression, and between‐species and within‐species between localities correlations between trait values measured in the field and greenhouse for different treatments.

Trait	Treatment	Major axis regression		Between‐species correlation		Within‐species between localities correlation	
		*N* = 16		*N* = 4		*N* = 16	
		*r*	*p*	*r*	*p*	*r*	*p*
SLA	CH	**.846**	**<.001**	.935	.065	**.534**	**.040**
	CL	**.597**	**.015**	.949	.051	.338	.209
	NH	**.636**	**.005**	.742	.258	.374	.162
	NL	**.732**	**.001**	.891	.109	.182	.505
LDMC	CH	.059	.829	.103	.897	.004	.989
	CL	.225	.401	.172	.828	.259	.339
	NH	.028	.917	.322	.678	.246	.364
	NL	.092	.735	.364	.636	.429	.106
Vegetative height	CH	**.517**	**.04**	.834	.166	.368	.169
	CL	**.663**	**.005**	.891	.109	.183	.501
	NH	**.533**	**.033**	.837	.163	.167	.540
	NL	.454	.077	.727	.273	.168	.537

*Note*: Statistically significant results (*p* < .05) are in bold.

Abbreviations: CH, competition and high water treatment; CL, competition and low water treatment; NH, no‐competition and high water treatment; NL, no‐competition and low water treatment.

## 4 DISCUSSION

In our greenhouse experiment, the most important differences in functional traits of closely related species were caused by species identity. Nevertheless, in experimental treatments, competition exerted a very important effect that, for some functional traits, even exceeded the variability caused by the species identity. Only in a few cases, there were differences in individual species' traits responses to experimental treatments (i.e., very few species by treatment interactions). Interspecific differences explained the most variability in clonal spread and SLA, and the least in LDMC and Aboveground/Belowground biomass compared to intraspecific differences. Values of some traits were closely correlated between greenhouse and field populations (e.g., SLA), while others were not (e.g., LDMC).

### 4.1 Inter‐ and intraspecific trait variability

All functional traits differed among our closely related species (subgenus *Carex* within the *Carex* genus), and, in most cases, species identity was the most important explanatory factor for overall trait variability. This is consistent with multiple studies (Dostál et al., 2020; Jung et al., 2014) that confirmed the importance of BTV, and also some in the case of phylogenetically closely related species (Diáz et al., 2013; Schmidt et al., 2018; Tammaru et al., 2021). Nevertheless, the effect of experimental treatments, particularly competition, and to a lesser extent source locality, was not negligible. In our study, the differences between BTV and ITV (both quantified by explained variability and by *F*‐values) varied among individual traits, which is in accordance with those mentioned in other studies (Garnier et al., 2001; Westerband et al., 2021). Clonal spread was the most varied across species in our experiment, with relatively low ITV. While Zhang et al. (2023) suggest high plasticity of clonal traits, our results confirm rather fixed interspecific differences with little ITV in clonal traits, similarly to Cornelissen et al. (2014). Whereas plant vegetative height, SLA or LDMC are the most frequently used traits for understanding the functional variation across species, our study showed that these traits can be at least as variable within species, or even more variable in the case of LDMC. In addition, BTV and ITV also differed among traits expressing similar function—for the Root/Shoot ratio considering only root biomass, the variability of BTV and ITV was similar, but if we also considered rhizomes (Aboveground/Belowground biomass), species specificity nearly disappeared. This result points to the necessity to address the inclusion of other belowground parts than roots to Aboveground / Belowground Biomass ratios. Importantly, it has been suggested that Root/Shoot ratio is rather plastic (e.g., Siefert et al., 2015), while it is not always clear if reported values include rhizome biomass or not.

However, it is of note that explained variability (characterized by Sum of Squares) is dependent on degrees of freedom and that ITV uses considerably more degrees of freedom (3 df for BTV represented by Species vs. 60 for ITV represented by source locality, competition, water availability and all interactions). There was also high Residual variability (i.e., variability among individuals under the same treatment and from the same locality), which was higher for SLA and vegetative height in the greenhouse experiment (i.e., under the controlled conditions rather than in the field). This might be specifically due to the fact that all experimental individuals were measured in the greenhouse, while individuals were selected according to Pérez‐Harguindeguy et al. (2016) in the field (i.e., excluding apparently unhealthy or damaged individuals); in actuality, truly random selection of individuals in the field is hardly practicable and even undesirable (see discussion in Mudrák et al., 2019).

From the two experimental treatments, which caused a substantial portion of ITV, competition had a more pronounced effect on trait values than water availability (though we acknowledge that the effect size is dependent on the intensity of the experimental manipulations). Competition affected all the traits, except Root/Shoot ratio; for LDMC, its effect was even greater than species identity. Tammaru et al. (2021) also found a significant effect of competition on *Carex* species, stronger than the effect of fertilization. Contrarily, Mudrák et al. (2019) showed that the species identity explained most of the variation in traits (mainly vegetative height, LDMC, and SLA) in a field experiment with fertilization and mowing. However, they used a wide range of species (generally all species present in grassland plots) such that BTV was much higher. Although we can expect that fertilization and mowing in this field experiment modified the competition strength, we can hardly compare their effect with our manipulation of competition, where we had either full competition, or individuals completely without competition.

In our experiment, competition shifted both SLA and LDMC from acquisitive toward more conservative values (i.e., lower SLA and higher LDMC). Zanzottera et al. (2020) showed a similar trend with high LDMC and low SLA for later successional stages with intensive competition, and the opposite pattern for early succession stages with low competitive pressure. Clonal spread was higher in the treatment without competition in our experiment. This was also confirmed by field studies where the number of clonal organs was higher in competition free gaps than in vegetation (Chaloupecká & Lepš, 2003; Macek & Lepš, 2003) because in competition free space fast vegetative spread is an advantageous strategy, while in highly competitive environments plants invest into seed production to escape either in space or in time (Chaloupecká & Lepš, 2003). While in all these cases the clonal spread was characterized by the number of ramets formed, competition often induces an increased length of clonal spacers (de Kroon & Hutchings, 1995); the relative magnitude of ITV will thus differ considerably among individual clonal traits.

Liu et al. (2017) and Dostál et al. (2020) demonstrated a significant effect of water stress on SLA, whereas in our study, water availability played a minor role. We can expect that a stronger drought treatment might have had stronger effects, but we decided for a less extreme contrast to not eliminate plants and still be able to measure the desired traits. Nevertheless, even small changes in water regime can play an important role in species communities (Leyer, 2005; Toogood et al., 2008). This was confirmed in our study by significant decrease of biomass of planted *Carex* species by 18%–24% in low water treatment. On the other hand, we did not measure any root traits (only root and rhizome biomass) that could react to the water treatment more significantly than aboveground traits (Taseski et al., 2021). Interestingly, we found a significant interaction between treatments only for LDMC, where water only played a role in the absence of competition. Other studies have showed inverse results where the effect of water availability was more significant in competition (Hao et al., 2023; Janíková & Lepš, 2023; Toogood et al., 2008).

Among possible biotic interactions, competition with dominant species might be the most important factor affecting survival of subordinate species, a role often occupied by the here studied *Carex* species. This competition effect varies not only spatially (fine grain heterogeneity), but also temporarily. If individual closely related species react to variation of this factor differently (shown by significant species × competition interaction), this might have positive effects on the coexistence of these closely related species. In our study, species responded differently to competition in three measured traits out of six. The same proportion (but for different traits) was found for the species × water availability interaction. However, in all these cases, the interactions were rather weak (relatively low values of *F* in Table 1). Many other studies recorded more insignificant than significant interactions between species and treatment (Albert et al., 2010; Garnier et al., 2001; Tammaru et al., 2021) but it was not shown for closely related species.

In both the greenhouse experiment and field study, the effect of source locality was significant for most traits. However, their interpretation was different. While the effect of source locality was part of ITV in both, in the field study the effect of source locality included both the genetic/epigenetic properties of the population and the effect of any environmental variation. In the greenhouse experiment however, the environmental variation was controlled and therefore could not affect the traits. Thus, ITV in pot experiments should be the result of only genetic/epigenetic differences among populations (Gorné et al., 2021; Puy et al., 2021). However, as the source material was individual ramets taken directly from the field, we cannot exclude any maternal effect, possible diseases or differences in endobionts (or generally, any of either symbiotic or antagonistic organisms) that might theoretically affect performance of the individuals. Although the source locality significantly affected the majority of traits measured in the greenhouse, its effect was always smaller than the effect of species identity and, except for Root/Shoot ratio, of competition. In the case of traits measured in the field, the effect of the source locality was also lower than the effect of species but was more impactful than in the greenhouse. We can thus assume that an important component of ITV in the field was impacted by environmental conditions. Other studies have shown the importance of both phenotypic and genotypic plasticity in ITV (Schmidt et al., 2018). For example, Gorné et al. (2021) prioritized genetic constraints as the main determinants of differences in traits of the leaf economic spectrum even within a species, while Donovan et al. (2011) highlighted more the phenotypic plasticity. It is quite difficult to understand the relative contributions of these determinants (Siefert et al., 2015), and more complex experiments with the inclusion of genetic methods (Puy et al., 2021) will be needed in the future to disentangle them and their contribution to assembly processes.

### 4.2 Correlations of traits between greenhouse experiment and field study

In our study, we also compared the correlations of functional traits between experimental (i.e., greenhouse experiment) and field conditions. Our results showed that while SLA correlated well, vegetative height correlated only weakly and LDMC did not correlate at all. In contrast to our results, Dostál et al. (2020), using a much wider range of species, showed in their review very significant correlations of both SLA and LDMC, but insignificant in the case of plant height across garden and field measurements. Our results comparing between‐species and within‐species effects confirm that SLA and vegetative height are rather species‐specific traits, and thus traits of even closely related species can correlate well in different ecological conditions, similarly as in former studies (Diáz et al., 2013; Schmidt et al., 2018).

Within‐species trends of the comparison of greenhouse and field conditions have shown a very weak relationship (significant just once out of 12 comparisons, with *p* = .04, which is close to Type I error). Interestingly, the only significant positive relationship was for SLA showing also otherwise high consistency (among species), and in the most realistic experimental conditions (competition and high water treatment). Our results show that the differences between populations were not reflected in the experiment because traits were importantly influenced by the experimental treatments while source locality explained only a quite small proportion of the variability in the greenhouse. This shows that while the differences between species are consistent under various experimental conditions, ITV among individual populations found in the field is clearly caused mainly by phenotypic plasticity, whereas genetic differences, if any, are rather small. Nevertheless, it is necessary to be very cautious when interpreting results obtained under highly controlled experimental conditions (e.g., greenhouse, climatic rooms, or garden experiments) and comparing them to those obtained under natural field conditions, as the results need not be always the same (Dostál et al., 2020; Tammaru et al., 2021).

## 5 CONCLUSION

Our study found that closely related species that are similar in their overall appearance and habitus can differ in their functional traits. At the same time, for some traits, phenotypic plasticity (particularly the part induced by competition) might exceed interspecific differences. Under standardized conditions, intraspecific differences caused by different source localities of individuals were sometimes significant, but considerably smaller than both BTV and competition‐induced phenotypic plasticity. There were large differences among individual traits in their magnitude of ITV compared to BTV. Surprisingly, the clonal spread measured by the number of ramets formed from a single mother plant exhibited the most stable interspecific differences. Taken together, these results point to a potential role of ITV in small‐scale coexistence, in particular for cases where closely related subordinate species respond to competition from dominants.

## AUTHOR CONTRIBUTIONS

**Eva Janíková:** Conceptualization (equal); data curation (lead); formal analysis (lead); methodology (equal); visualization (lead); writing – original draft (lead). **Marie Konečná:** Conceptualization (equal); data curation (equal); methodology (equal). **Aleš Lisner:** Conceptualization (equal); data curation (equal); methodology (equal). **Markéta Applová:** Data curation (equal). **Petr Blažek:** Data curation (equal); methodology (supporting). **Anna E‐Vojtkó:** Data curation (equal). **Lars Götzenberger:** Conceptualization (equal); data curation (equal); funding acquisition (lead); methodology (equal); supervision (supporting). **Jan Lepš:** Conceptualization (lead); data curation (supporting); formal analysis (supporting); funding acquisition (lead); methodology (lead); supervision (lead).

## CONFLICT OF INTEREST STATEMENT

The authors declare no conflict of interest.

## Supporting information


Appendix S1.

## Data Availability

Upon acceptance, data will be stored in the Dryad Digital Repository. For reviewing process, data are accessible as supplementary information (file Data–for reviewers but not for publication).
