# Correction to “Closely Related Species Differ in Their Traits, but Competition Induces High Intra‐Specific Variability”

**DOI:** 10.1002/ece3.71017

**Published:** 2025-02-13

Janíková, E., Konečná, M., Lisner, A., Applová, M., Blažek, P., E‐Vojtkó, A., Götzenberger, L., & Lepš, J. (2024). Closely related species differ in their traits, but competition induces high intra‐specific variability. Ecology and Evolution 14:e70254. https://doi.org/10.1002/ece3.70254

In the “Data Availability Statement” section, the text about the storage of data “Upon acceptance, data will be stored in the Dryad Digital Repository. For reviewing process, data are accessible as supplementary information (file Data—for reviewers but not for publication).” was corrected to “Data are stored in the Dryad Digital Repository https://datadryad.org/stash/dashboard?doi=10.5061%2Fdryad.69p8cz9bh.”

We apologize for this error.

For all the authors,

Eva Janíková

