# Supplementary material for: Closely related species differ in their traits, but competition induces high intra‐specific variability
Source: Ecol Evol. 2024 Sep 13;14(9):e70254. doi: 10.1002/ece3.70254 (PMC11393774; doi:10.1002/ece3.70254)
Supplement: Supplementary file 1 — Appendix S1. [file ECE3-14-e70254-s001.docx]

**Appendix: Additional tables and figures**

Table S1: *Carex* species and Populations used in our experiment. 1 – population, 0 – no-population.

|  | **Bošice** | **Ohrazení** | **Tejmlov** | **Úhřice** | **Vrcov** | **Závraty** | **Zvíkov** |
| --- | --- | --- | --- | --- | --- | --- | --- |
|  | 49°5'N, 13°50'E | 48°57´N, 14°35´E | 49°7.9'N, 13°39'E | 49°4.7'N 13°55'E | 48°55´N, 14°39´E | 48°56´N, 14°23´E | 48°59´N, 14°36´E |
|  | 620 m a.s.l. | 510 m a.s.l. | 900 m a.s.l. | 650 m a.s.l. | 510 m a.s.l. | 460 m a.s.l. | 500 m a.s.l. |
| *C. caryophyllea* | 0 | 0 | 0 | 1 | 1 | 1 | 1 |
| *C. pallescens* | 1 | 1 | 0 | 1 | 1 | 0 | 0 |
| *C. panicea* | 1 | 1 | 1 | 0 | 1 | 0 | 0 |
| *C. pilulifera* | 0 | 1 | 1 | 1 | 1 | 0 | 0 |

Table S2: Results of the effect of factor Block. Type I ANOVA for linear model of individual traits measured in greenhouse experiment in the case that block is supposed to be fixed factor. Statistically significant results (p<0.05) are in bold.

|  | DF | F | p |
| --- | --- | --- | --- |
| SLA | **4, 242** | **4.06** | **0.003** |
| LDMC | **4, 243** | **7.38** | **<0.001** |
| Vegetative Height | 4, 248 | 0.41 | 0.799 |
| Clonal Spread | **4, 249** | **3.65** | **0.007** |
| Root/Shoot Ratio | 4, 245 | 1.26 | 0.288 |
| Aboveground/Belowground Biomass | 4, 244 | 0.52 | 0.718 |

Table S3: Results of ANOVA for linear model of individual functional traits measured in the field. Statistically significant results (p<0.05) are in bold.

|  |  |  | SLA | |  | LDMC | |  | Vegetative Height | |
| --- | --- | --- | --- | --- | --- | --- | --- | --- | --- | --- |
|  | DF |  | F | p |  | F | p |  | F | p |
| Species | 3, 155 |  | **48.51** | **<0.001** |  | **16.04** | **<0.001** |  | **74.21** | **<0.001** |
| Locality | 6, 149 |  | **6.03** | **<0.001** |  | **9.38** | **<0.001** |  | **16** | **<0.001** |
| Species*Locality | 6, 143 |  | 0.23 | 0.967 |  | 1.4 | 0.218 |  | **3.37** | **0.004** |


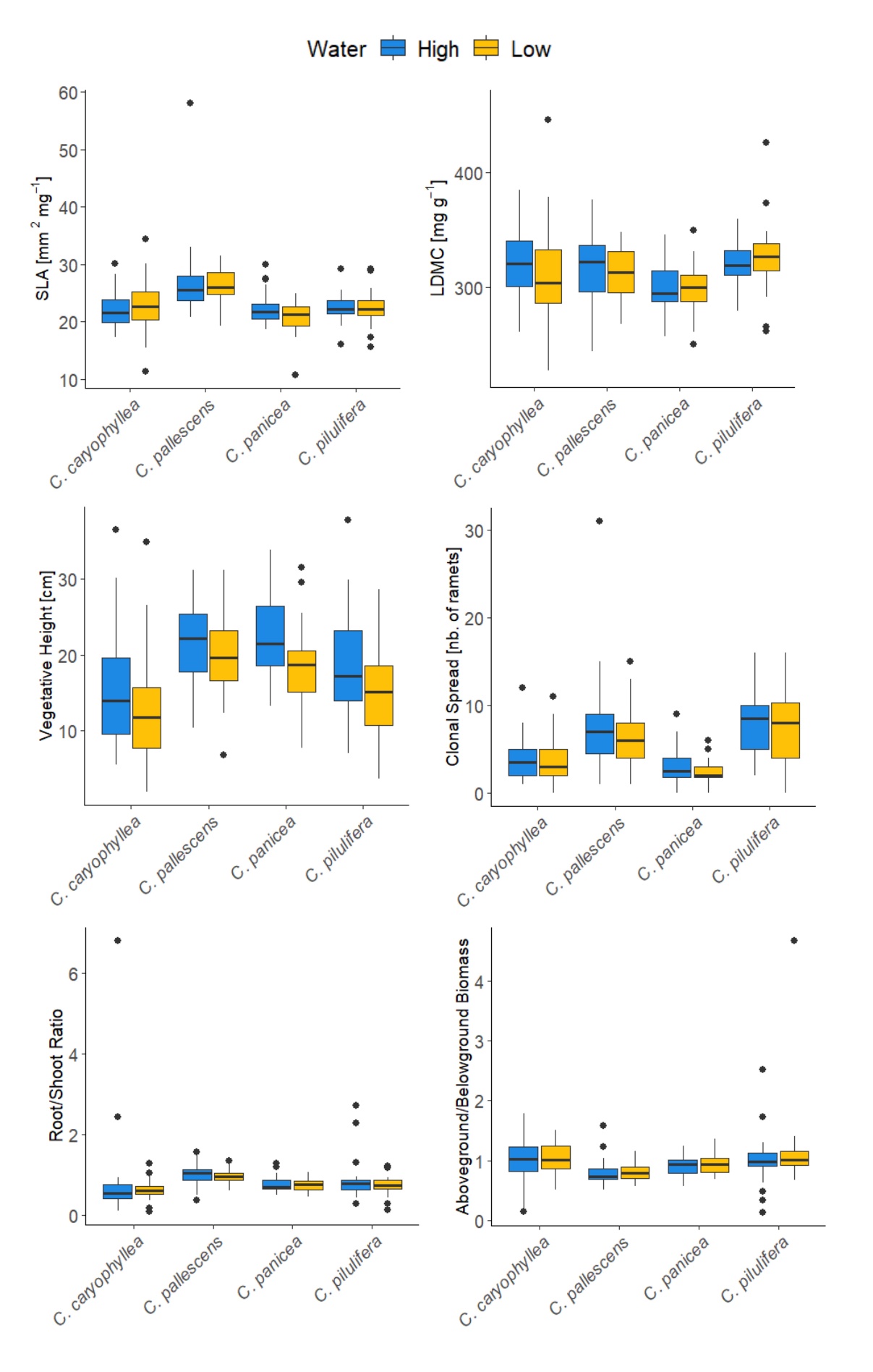


Figure S1: Species*water interaction of functional traits measured in greenhouse experiment. Results of statistical test are in Table 1 in the main text.

Figure S2: Statistically significant Competition*Water interaction for LDMC measured in greenhouse experiment. Result of statistical test is in Table 1 in the main text.
